# Study on the mechanical characteristics and anchorage performance of the anchor cable with precast internal-anchor-head (PIAC)

**DOI:** 10.1038/s41598-025-22107-x

**Published:** 2025-10-31

**Authors:** Bo Liu, Yufang Zhang, Kun Yuan, Jian Li, Jian Cui, Junli Wan, Jiawei Fan

**Affiliations:** 1 Railway Engineering Research Institute, China Academy of Railway Sciences Corporation Limited, Beijing, 100081 China; 2 National Key Laboratory of High-Speed Railway Track System, China Academy of Railway Sciences Corporation Limited, Beijing, 100081 China

**Keywords:** Precast internal-anchor-head (PIAH), Anchor cable structure, Theoretical analysis, Full-scale tension experiments, Numerical simulation, Engineering, Materials science

## Abstract

In order to improve the anchoring force of traditional anchor cables while ensuring low cost, this paper proposed a new-type anchor cable structure with a precast internal-anchor-head (PIAH) at the anchor cable end. The reliability of the new structure was thoroughly validated through theoretical analysis, indoor pull-out experiments, and numerical simulations. Firstly, the mechanical pattern based on the characteristics and anchoring mechanism of the PIAH structure was analyzed, and the calculation methods under different failure modes were proposed. Secondly, a full-scale physical model experiment was conducted to study the effects of varying lengths PIAH on the tensile stress of the grout, stress distribution between grouting sections and the surrounding rock, shear stress distribution between PIAH and grout, and the yield conditions of the PIAH. Results show that after applying the PIAH, the ultimate bearing capacity increases from 796.4 to 921.2 kN, and the ultimate strain decreases from -1219.7 × 10^–6^ to -309.5 × 10^–6^, a reduction of 74.63%. It indicates that the strain state of the grout will be substantially improved after setting the PIAH in anchor cable, and the bond strength between the grout and the hole wall could be enhanced, result in effective increase of anchoring force. At the same time, longer PIAH are more favorable for controlling cable deformation. Furthermore, to further validate the experimental results, numerical models of multiple-types anchor cable structures were established based on finite difference numerical method. Simulation results show that, under the same conditions, the ultimate bearing force of the anchor cable increases as the length of PIAH increases, and the overall elastic modulus of the anchoring system could also be improved. Compared to the grout of the Traditional Prestressed Anchor Cable (TPAC), the concentration degree of axial stress of Anchor Cable with Precast Internal-anchor-head (PIAC) was only 28.57%, effectively reduced the degree of stress concentration. Additionally, the maximum effective length of PIAH is about 1.5 ~ 2 m under the test conditions of a 4 m PIAC and further length increases produce diminishing returns. Meanwhile, PIAC can be used not only in hard rock formations, but also in soft rock formations if improving the strength of the interface between the surrounding rock and the grout. The research results have valuable reference significance for the engineering application of PIAC.

## Introduction

Prestressed anchor cables, consisting of anchor head, free section, and anchoring section, often serve as a fundamental support system in geotechnical engineering by applying compressive stress to rock masses and generating stabilizing stress fields in surrounding strata^1^. It has been widely used in slope protection, mining operations, underground construction and rock support due to the advantages of small excavation disturbance, high construction efficiency, and low cost^2–6^. In soft rock formations, corrosive formations, and formations with abundant groundwater, the Traditional Prestressed Anchor Cable (TPAC) may encounter challenges such as stress concentration and premature failure of the bottom pressure plate due to its reliance on the bonding force between the grout and surrounding rock. Therefore, it is imperative to implement enhancements to address the unique characteristics of these rock formations.

To optimize anchor cable performance in geotechnical engineering applications, a comprehensive understanding of their mechanical behavior under various loading conditions is crucial. Extensive experimental and theoretical investigations have been conducted on anchoring mechanisms, with particular focus on load-transfer characteristics and deformation responses^7–9^. Recent advancements include the development of sophisticated constitutive models that capture the complex interaction between anchors and surrounding rock masses, particularly in challenging geological conditions involving creep behavior^6,9^. Researchers have employed diverse methodologies to examine anchor performance, ranging from double shear experiments analyzing landslide impacts^10^ to numerical simulations investigating dynamic responses under seismic or blast loading scenarios^11,12^. Key findings from these studies demonstrate that anchor system effectiveness is governed by an intricate interplay of multiple factors, including prestress level and its temporal evolution^13,14^, environmental exposure conditions such as wet-dry cycling^12^, geometric parameters like anchor length and diameter^15,16^, and installation quality considerations^17^. Despite these advancements, meaningful challenges persist in practical applications, particularly concerning stress distribution non-uniformity leading to localized overstress zones^18^, accurate measurement and interpretation of service loads^19,20^, and long-term performance degradation mechanisms in aggressive environments^12^. These unresolved issues underscore the pressing need for further systematic investigation into optimized anchor configurations, and enhanced monitoring techniques to ensure long-term structural integrity in critical infrastructure projects.

Recent advancements in anchor cable technology have led to the development of various enhanced anchor cable structures, including Fiber-Reinforced Polymer (FRP) cables^21^, self-anchored Carbon Fiber Reinforced Polymer (CFRP) anchor cables^22,23^, and NPR anchor cables^24,25^, each offering unique mechanical advantages but facing limitations in practical implementation. While these innovations demonstrate improved performance characteristics, such as the superior creep resistance of CFRP anchor cable and the remarkable elongation capacity (45.2%) and constant resistance (650kN) of NPR cables^26^, their widespread adoption is constrained by significant drawbacks including high material costs, structural complexity^27^, and the CFRP anchor cable is susceptibility to brittle failure requiring specialized fixtures. In this paper, the Anchor Cable with Precast Internal-anchor-head (PIAC) emerges as a particularly promising solution, combining superior engineering performance with better economic feasibility. Compared to conventional TPAC and above structure, PIAC demonstrates enhanced load-bearing capacity through its elongated Precast Internal-Anchor-Head (PIAH), which effectively controls deformation while improving bond strength at the grout-rock interface. The optimized strain distribution of PIAC not only increases ultimate bearing capacity but also noticeably improves shear and slip resistance, mitigating stress concentration and reducing local peak strains. These technical advantages, coupled with the relatively simple structure and lower implementation costs compared to NPR and CFRP alternatives, make it particularly suitable for large-scale engineering applications where both performance and cost-effectiveness are critical considerations, offering a balanced solution that addresses the key limitations of existing advanced anchor cables while maintaining practical constructability.

Building upon previous advancements in anchor cable technology, this study introduces an innovative PIAC structure to address critical gaps in current anchoring mechanisms. The research methodology systematically investigates the PIAC structure through three key phases including theoretical analysis, experimental validation, and numerical simulation. Initially, the mechanical behavior and structural performance of the PIAC are rigorously analyzed through theoretical modeling, establishing fundamental calculation methods based on its unique structural configuration and anchoring principles. The experimental phase employs a comprehensive full-scale testing apparatus to evaluate the structural reliability of PIAC and quantify the relationship between anchor head length and anchoring efficiency. Then, sophisticated numerical simulations replicate the experimental conditions, providing detailed insights into the influence on key performance parameters including cable displacement patterns, axial stress distribution within the grouting medium, interfacial stress characteristics at the grout-rock interface, and yield progression in the grout ahead of the anchor head. Finally, we discussed the relative errors between experimental and simulation results, maximum effective length of PIAH, geotechnical applicability of PIAC structures and the limitations and future research directions. These integrated investigations not only advance the fundamental understanding of prestressed anchoring mechanisms but also establish a robust technical foundation for optimizing anchor cable design in practical engineering applications. The multi-methodological approach ensures both theoretical rigor and practical relevance, offering meaningful contributions to the field of geotechnical anchoring technology.

## The structural characteristics of PIAC

Anchor cables have been extensively employed in slope and tunnel reinforcement projects worldwide for decades. Based on their load-transfer mechanisms, prestressed anchor cables can be classified into pressure-dispersed type and tension-concentrated type^28,29^. Tension-concentrated type cables, featuring fully bonded steel strands along the grouted section, are primarily suitable for intact rock masses, though their stress concentration near the loading point often leads to grout tensile cracking^30^. In contrast, pressure-dispersed cables utilize unbonded strands that transfer tension to grout through bearing plates at the anchor base, converting tensile forces into compressive stresses. The development of pressure-dispersed anchors with multiple bearing plates further enhances load distribution and geological adaptability, making them widely adopted in practice^31^.

This study focuses on a novel prestressed cable system, an advanced pressure-dispersed type variant that replaces conventional bearing plates with PIAH. The PIAC structure uniquely transforms strand tension into both grout compression and shear resistance at the PIAH-grout interface, noticeably improving anchoring performance. Compared to traditional dispersive pressure type anchors, it introduces a new high-strength concrete-to-concrete interface capable of substantial shear resistance. This structural innovation redistributes concentrated loads into distributed stresses, with its mechanical behavior primarily governed by the precast head length because the borehole dimensions is usually fixed. While demonstrating promising field performance, the structure lacks experimental validation. Therefore, this research systematically investigates the mechanical characteristics of PIAC through full-scale physical modeling, examining how PIAH length influences grout tensile stress, grout-rock interface stress distribution, PIAH-grout shear stress distribution, and grout yielding conditions, with numerical simulations employed to verify experimental findings.

### Structure style

**Fig. 1:**
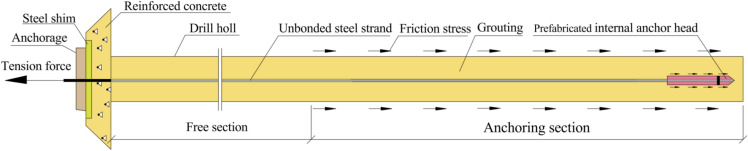
The Schematic diagram of the structural composition and force mode of the PIAC.

The PIAC structure represents an enhanced anchor cable structure derived from the TPAC. By incorporating a PIAH at the end of the anchoring section of TPAC, the anchoring effect can be substantially improved. As shown in Fig. 1, the structure of the PIAC primarily consists of PIAH, grout, unbonded steel strands, reinforced concrete piers, steel bearing plate, and anchorages, with PIAH serving as the central component of the structure.

**Fig. 2:**
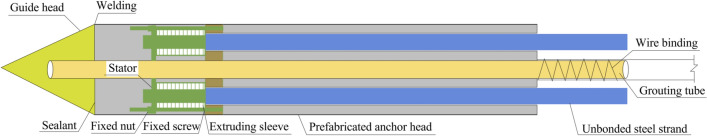
The schematic diagram of the structure of the PIAH in PIAC.

As depicted in Fig. 2, the PIAH is a precast concrete component, manufactured by incorporating grouting pipes and unbound steel strands. Additionally, a guide cap is welded at the leading edge and linked to the anchoring body of the anchoring section to facilitate the transmission of the anchoring force. After applying prestress to the unbound steel strand, the force is successively transmitted to the steel bearing plate, high-strength PIAH, and the joint between the grout and the surrounding rock, resulting in a substantially reduction in compressive stress on the anchor head. In contrast, for anchor heads of TPAC, only the columnar grout at the front edge is responsible for transmitting the anchoring force, leading to elevated local stress in the contact region. Consequently, the innovative anchor cable structure of PIAC notably enhances the stress distribution within the anchoring system.

### Structure characteristic

By incorporating a PIAH at the termination of the anchoring section of the PIAC, the structural integrity of the anchor cable has been enhanced, leading to notable alterations in the mechanical pattern. The primary structural characteristics are outlined as follows:

1. In contrast to TPAC, the PIAC boasts a larger contact area with the grout, facilitating enhanced transmission of anchoring forces and substantially bolstering the anchoring force of anchor cable.
2. Utilizing high-strength concrete to cast steel strands, extruded sleeves, and bearing plates as a unified unit enables robust load-bearing capacity and ensures the durability of the bearing body. This approach not only enhances the load-bearing capacity but also safeguards the steel strands from corrosion, thereby extending the longevity of the anchor system.

## Failure mode and calculation method

### Mechanical pattern

**Fig. 3:**
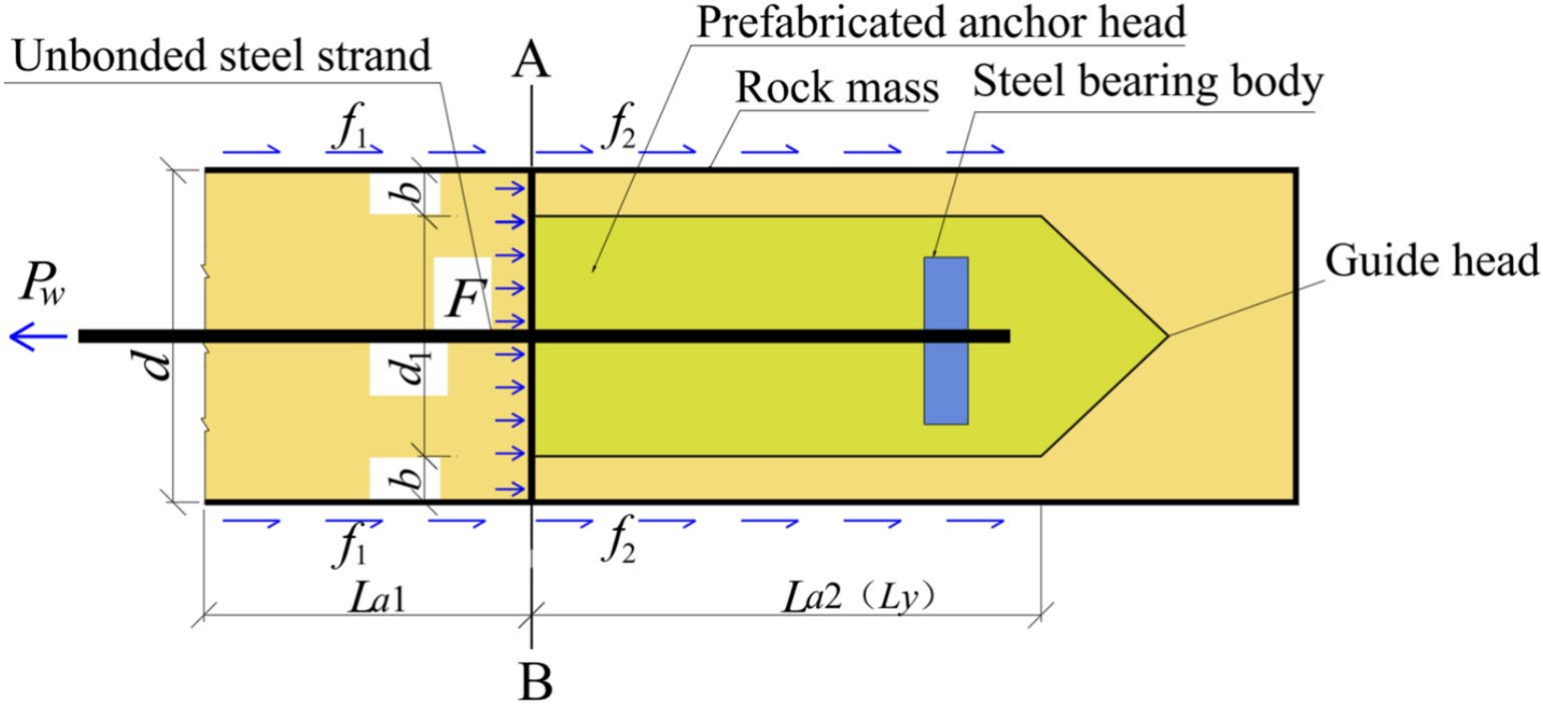
The schematic diagram of the force mode of PIAC.

As a rigid body, the PIAH interacts with the surrounding grout, transmitting part of the anchoring force to the surrounding rock mass. The surrounding rock mass provides frictional force on the ${L}_{y}({L}_{a2})$ section, ${f}_{2}$ is the residual anchoring force, and the force *F* will be overcome by the compressive stress on the AB circular section. The friction on the surface of the hole wall above AB surface provides friction *f*_1_, which is equal to *f*. Therefore, *P*_*w*_ = *F* + *f*_2_ = *f*_1_ + *f*_2_. The force characteristics of PIAC is shown in Fig. 3.

The failure modes of PIAC are as follows:

#### Compression failure on AB surface

Due to excessive local compressive stress on the AB surface, the grout suffered from compression failure. Compared with TPAC, the compression range is only in the *d*_*1*_ area inside the bearing plate, so the circular grout within the *Ly* range greatly increases the bearing area.

#### Slip failure at hole wall

After fully utilizing the frictional resistance *f*_*1*_ of the precast anchor head section, the frictional resistance of the anchor sections above AB surface plays a similar role to that of traditional precast anchor heads. When *f*_*1*_ is large enough, the precast anchor head will be damaged. Compared with traditional pressure anchor cables, *f*_*1*_ is less than the anchor cable tension *P*_*w*_, and the *P*_*w*_ of TPAC is completely overcome by *f*_*1*_, where *P*_*w*_ is equal to *f*_*1*_.

### Calculation method

#### Compression failure on AB surface

Under the condition of compression failure on AB surface, the ultimate load of PIAH is calculated according to the following formula:

1 $${P}_{w}=F+{f}_{2}=\frac{\pi }{4}\left({d}^{2}-4{d}_{g}^{2}\right)R\eta +\pi d{L}_{a2}{f}_{rb2}{\varphi }_{2}$$

${P}_{w}$ is the calculated ultimate load of PIAH, kN; *d* is the diameter of anchor section, m; *d*_*R*_ is the diameter of anchor body, m; *R* is the unconfined axial compressive strength of the PIAH, N/mm^2^; $\eta$ is the amplification factor of the transverse axial compressive strength of the precast anchor head determined through experiments, ${L}_{y}({L}_{a2})$ is the axial length of the PIAH, m; ${f}_{rb2}$ is the friction resistance between grout, N/mm^2^; ${\varphi }_{2}$ is the influence coefficient of the length of the PIAH anchorage segment on the bond strength, which is equal to 1.6.

#### Slip failure at hole wall

The ultimate load of PIAH under the second failure mode is calculated according to the following equation:

2 $${P}_{w}={f}_{1}+{f}_{2}=\pi d{L}_{a1}{f}_{rb1}{\varphi }_{1}+\pi d{L}_{a2}{f}_{rb2}{\varphi }_{2}$$

${L}_{a1}$ is the length of front section of PIAH, m; ${f}_{rb1}$ is the bond strength between grout and hole wall of anchor section, N/mm^2^, the values are shown as Table 1; ${f}_{rb2}$ is the bond strength between grout and the hole wall of the PIAH, N/mm^2^, the values are shown as Table 2; ${\varphi }_{1}$ is the influence coefficient of anchorage length on bond strength, the values are shown as Table 3. Take the smaller value calculated by Eqs. (1) and (2) as the ultimate load of the prestressed anchor cable. Tables 1, 2, 3 are from “Specifications for Design of Highway Subgrades”^32^ in China.

**Table 1:** The bond strength *f*_*rb1*_ between grout and hole wall of anchor section.

Rock mass type	Bond strength *f*_*rb1*_ (kPa)	Rock mass type	Bond strength *f*_*rb1*_ (kPa)
Extremely soft rock	135 ~ 180	Harder rock	550 ~ 900
Soft rock	180 ~ 380	Hard rock	900 ~ 1300
Softer rock	380 ~ 550

**Table 2:** The bond strength *f*_*rb2*_ between grout and the hole wall of PIAH*.*

Soil type	The state of soil	Bond strength *f*_*rb2*_ (kPa)
Cohesive soil	Hard	32 ~ 40
	Hard plastic	25 ~ 32
	Soft plastic	15 ~ 20
Sandy soil	Loose	30 ~ 50
	Slightly dense	50 ~ 70
	Medium dense	70 ~ 105
	Dense	105 ~ 140
Gravel soil	Slightly dense	60 ~ 90
	Medium dense	80 ~ 110
	dense	110 ~ 150

**Table 3:** The influence coefficient of anchorage length on bond strength.

Rock mass type	Anchorage length (m)	Value of *φ*_*1*_	Rock mass type	Anchorage length (m)	Value of *φ*_*2*_
Soil	13 ~ 16	0.8 ~ 0.6	Soft or extremely soft rock	9 ~ 12	0.8 ~ 0.6
	10 ~ 13	1.0 ~ 0.8		6 ~ 9	1.0 ~ 0.8
	10.0	1.0		6.0	1.0
	10 ~ 6	1.0 ~ 1.3		6 ~ 4	1.0 ~ 1.3
	6 ~ 3	1.3 ~ 1.6		4 ~ 2	1.3 ~ 1.6

### Anchorage length

The anchorage length of the anchor cable is determined by the following formula:

3 $${L}_{a}={L}_{a1}+{L}_{a2}=\frac{{K}_{2}{P}_{d}}{\pi d{f}_{rb1}{\varphi }_{1}}-\frac{{f}_{rb2}{\varphi }_{2}{L}_{a2}}{{f}_{rb1}{\varphi }_{1}}+{L}_{a2}$$

${K}_{2}$ is th safety factor; $\xi$ is the reduction factor of interface bonding strength, with a value ranging from 0.6 to 0.85; ${f}_{b}$ is the friction resistance between of grout and hole wall, N/mm^2^.

### Area of bearing plate

The area of the bearing plate needs to ensure that the grout could not be crushed, and the calculation formula is as follows:

4 $${K}_{p}({P}_{d}-\pi d{f}_{rb2}{\varphi }_{2}{L}_{a2})\le 1.35{A}_{p}\eta {f}_{c}$$

${A}_{p}$ is the net contact area between the anchor cable and the grouting section of the anchoring section, which is the area of the compression zone, mm^2^; ${K}_{p}$ is the local compression safety factor of grout, which can be set to 2.0; ${f}_{c}$ is standard value for the axial compression strength of grout, N/mm^2^.

## Pull-out experiments of PIAC

To investigate the impact of varying PIAH lengths on the anchoring effect of PIAC, four groups of indoor pull-out experiments were devised. Among them, the group 1 is the control group, which uses TPAC. Meanwhile, Groups 2 to 4 served as experimental groups, employing PIAC with the internal-anchor-head lengths of 250, 500, and 1000 mm. The experiments were respectively named TPAC, PIAH250, PIAH 500, and PIAH 1000.

### Test model

The production process of full-size indoor testing model of PIAC includes PIAH production, sensor installation, and concrete pier pouring.

#### PIAH production

**Fig. 4:**
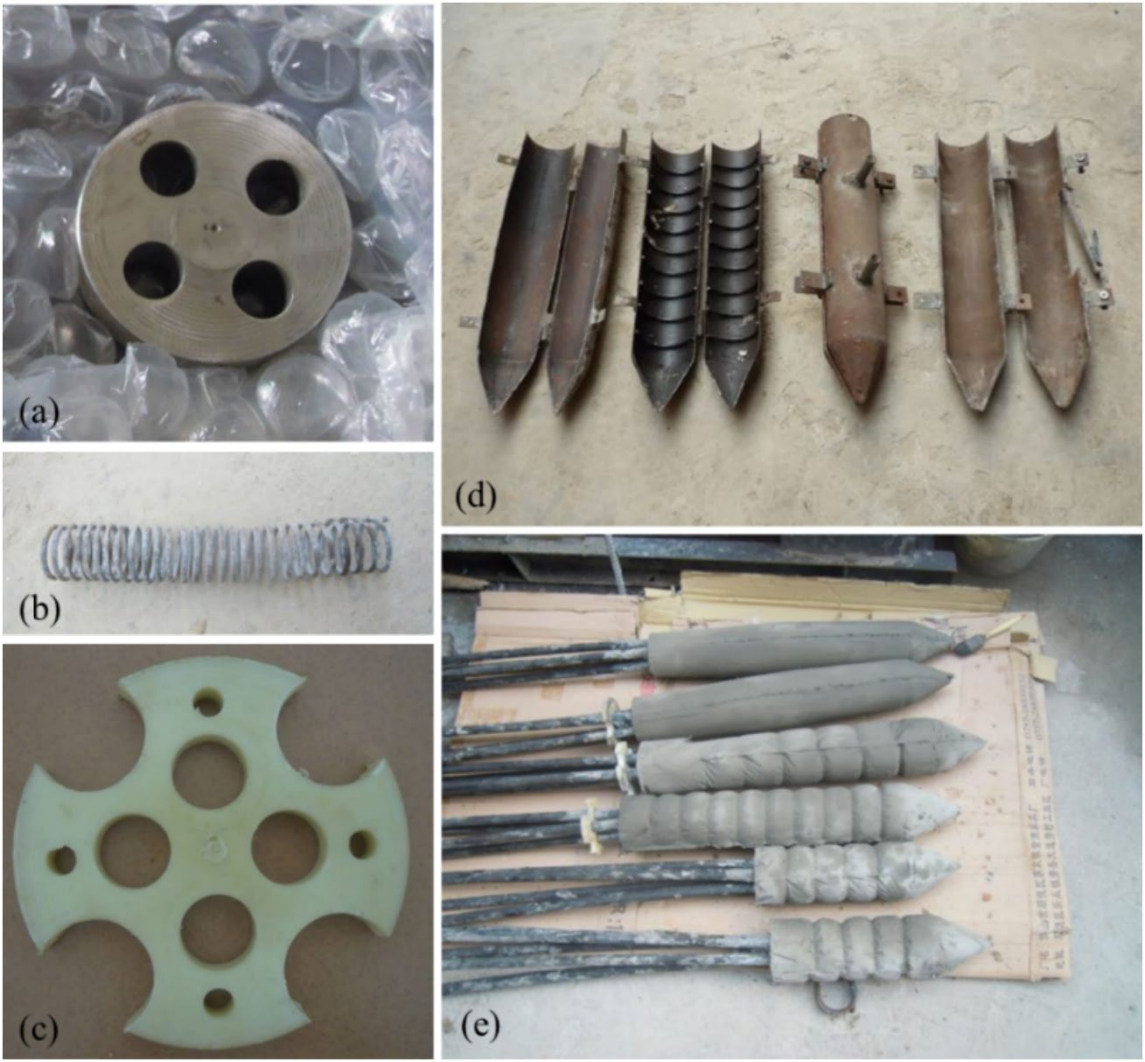
PIAC samples. (**a**) Steel bearing plate; (**b**) Spiral reinforcement ribs; (**c**) Wire ring; (**d**) Mold; (**e**) PIAC.

The high strength concrete PIAH primarily consists of high-strength concrete, steel bearing plates, spiral stirrups, and wire rings. The high strength concrete is composed of fiber-reinforced fine aggregate concrete, renowned for its exceptional strength and crack resistance. Anchor holes are accommodated on the steel pressure plate, with the structure of the pressure plate illustrated in Fig. 4a. Spiral reinforcement is mainly used to improve the compressive strength of PIAH (Fig. 4b). The wire ring is processed with standardized nylon rods, and the anchor holes on the wire ring correspond to the anchor holes on the bearing plate. Grouting holes and positioning holes are reserved around the perimeter (Fig. 4c). The high-strength concrete PIAH needs to be precast using a set mold (Fig. 4d), and the actual high-strength concrete PIAH are shown in Fig. 4e. In this physical model test, the utilized anchor cable was composed of 4 steel strands, each with a diameter of 15.2 mm. The total length of the anchor cable was 4 m.

#### Surrounding rock fabrication

**Fig. 5:**
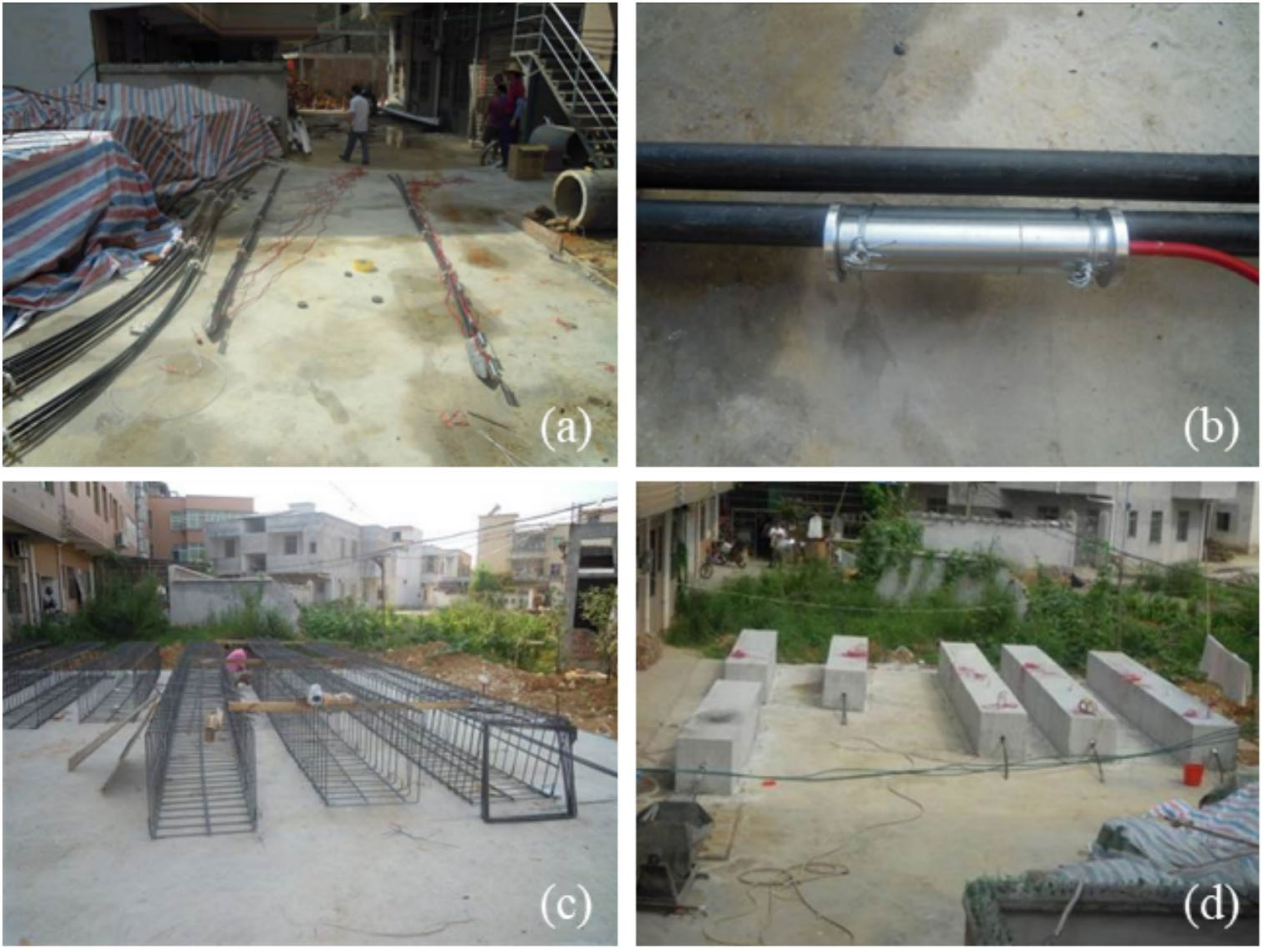
Sensor installation and concrete pier pouring process. (**a**) Anchor cables with PIAH; (**b**) Installation of strain gauges; (**c**) Steel mesh production; (**d**) Completed test piece after pouring with PIAC.

The same model size, materials, and experimental conditions were used for each group of experiments, as shown in Fig. 5. The model size is 5000 mm × 800 mm × 800 mm, and the material used is C40 concrete to simulate hard rock mass. The grout had a length of 4500 mm, and the borehole diameter was 150 mm. The testing equipment are shown in Table 4, mainly including drawing equipment, matching locks, testing instruments, etc., as shown in Fig. 5.

**Table 4:** List of measurement equipment in full-scale tension experiments.

Number	Name	Model	Unit	Quantity
1	Lifting jack	YCW150B-200	PCS	1
2	The jack supports the foot	YCW150B	PCS	1
3	Electric pump	ZB4-500	PCS	1
4	Manual oil pump	ZY-100	PCS	1
5	Extrusion machine	GYJC50-150	PCS	1
6	Four-beam tool anchor	OVM15-4G	set	1
8	Tool clamping piece	OVM15G	set	1
9	Four limit plate	OVM15-4G	PCS	1
11	Multipoint centralized Measurement of microcomputer	CUD-3U	set	1
12	Software processing system	CUD-3U	set	1
13	Embedded concrete strain gauge	TGCL-2-100A	PCS	32
14	Vibrating string anchor cable dynamometer	TGCL-3–200	PCS	1

#### Installation of sensors

**Fig. 6:**
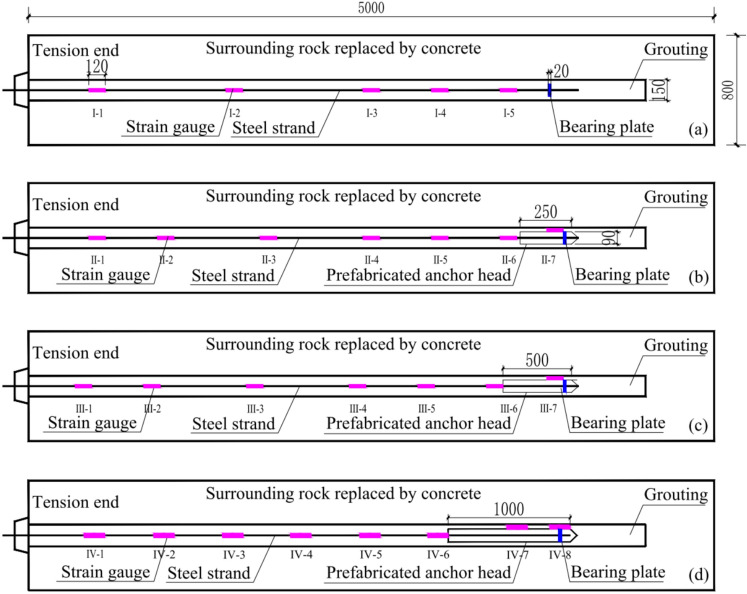
Layout of anchor cable strain gauges for full-size model testing. (**a**) TPAC; (**b**) PIAH250; (**c**) PIAH 500; (**d**) PIAH 1000.

The experimental testing primarily involves the strain of the grout and the anchoring force of the anchor cable. The anchoring force of the anchor cable can be measured by the vibrating string type anchor cable dynamometer at the anchor device. Multiple strain gauges and an anchoring force meter are arranged along the bearing plate from a compact to sparse configuration, enabling the recording of stress and strain data throughout the testing procedure. The layout of strain gauges in various experimental groups is depicted Fig. 6.

### Experimental procedure

Prior to commencing the experiment, set up various data monitoring instruments and conduct preliminary testing and calibration to verify the reliability of the data. During the experiment, employ a jack to apply cyclic loads to the structure under different design conditions, concurrently recording the surface strain of the PIAH at each load level. The loading speed in the early stage should not exceed 100 kN/min, and should not exceed 50 kN/min in the later stage. Observations should be made per 100 kN. After loading to a certain level, the load should be completely unloaded before reloading to the next level, and the loading should be repeated step by step. The maximum tension load is 900kN. The observation time for each level is 5 min. Considering that failure may occur after the load reaches 70% of the ultimate load of the steel strand, in order to accurately measure the ultimate load of the anchor cable, the loading increment should be appropriately reduced and the loading rate should be slowed down according to the site conditions. When the following situations occur in the experiment, it can be determined that the anchor cable is damaged: (1) The anchor body is pulled out of the surrounding rock or the steel strand is pulled out or broken from the anchor body; (2) The displacement of anchor head does not converge; (3) The displacement increment of the anchor head resulting from the subsequent load must equal or exceed twice the displacement increment of the prior load.

Owing to budgetary constraints inherent in the experimental process, the sample number for each test group was limited to three, with identical testing procedures applied to each specimen within a group. Given this limited replication, analysis of variance (ANOVA) would be statistically underpowered and associated with an unacceptably high risk of false negatives. Consequently, the averaging method commonly employed in physical experimentation was adopted. This analytical approach commenced with an assessment of data consistency within each triplet set by calculating the mean and standard deviation. Potential outliers were identified and subsequently subjected to Grubbs’ test for statistical validation. Only datasets confirming to the assumption of normality and containing no statistically identified outliers were deemed suitable for mean value representation.

### Result

#### Load – displacement analysis

**Fig. 7:**
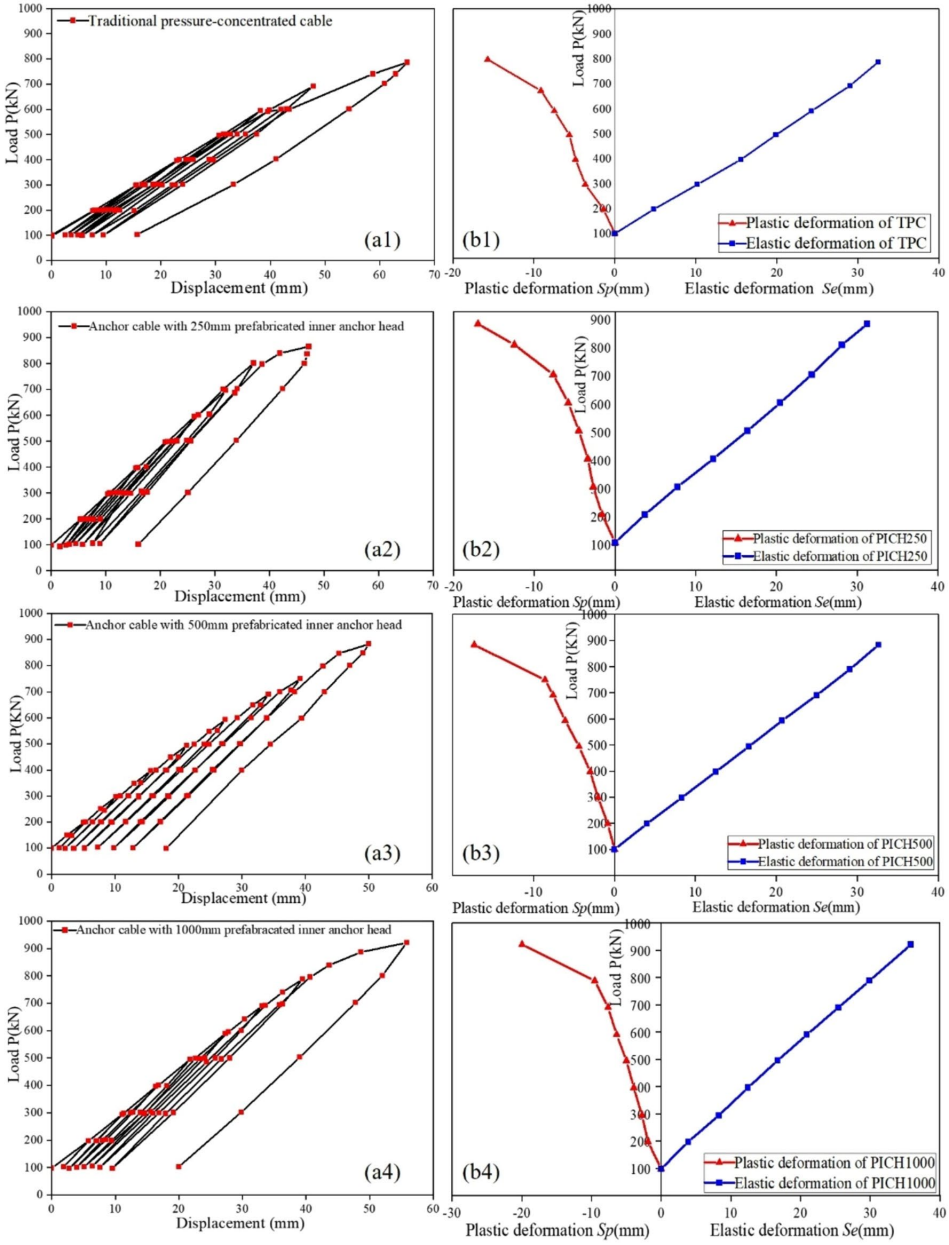
Load—displacement curves and elastic–plastic displacement characteristics of anchor cables under different working conditions. (**a1** and **b1**) TPAC; (**a2** and **b2**) PIAH250; (**a3** and **b3**) PIAH 500; a4 and b4. PIAH 1000.

The load–displacement curves of the anchor cables under four working conditions are shown in Fig. 7a1–a4. Through cyclic loading experiments, it was found that the ultimate bearing capacity of TPAC in the pull-out experiment reached 796.4 kN, while the anchor cables using 250, 500, and 1000 mm PIAH had ultimate bearing capacities of 865.5, 883.6, and 921.2 kN, respectively. It indicates that the use of PIAH substantially improves the ultimate anchoring force of the anchor cables. The maximum displacements of four groups are 15 mm, 16 mm, 18 mm, and 20 mm, respectively, with a relatively small increase in deformation.

The displacement of the anchor cable consists of elastic displacement and plastic displacement. As shown in Fig. 7b1–b4, during the tension stage, the elastic displacement increases linearly in all four working conditions, but the plastic displacement has an obvious inflection point. When the load exceeds the inflection point value, the plastic displacement increases rapidly, indicating that the anchor cable will gradually fail and break. The inflection points of the four working conditions are 671.9, 695.9, 749.7, and 788.4 kN, indicating that due to the setting of the PIAH, the maximum bearing capacity of the anchor cable is increased. At the same time, the longer the PIAH, the stronger its ultimate bearing capacity.

#### Strain analysis of different parts of the anchor cable

**Fig. 8:**
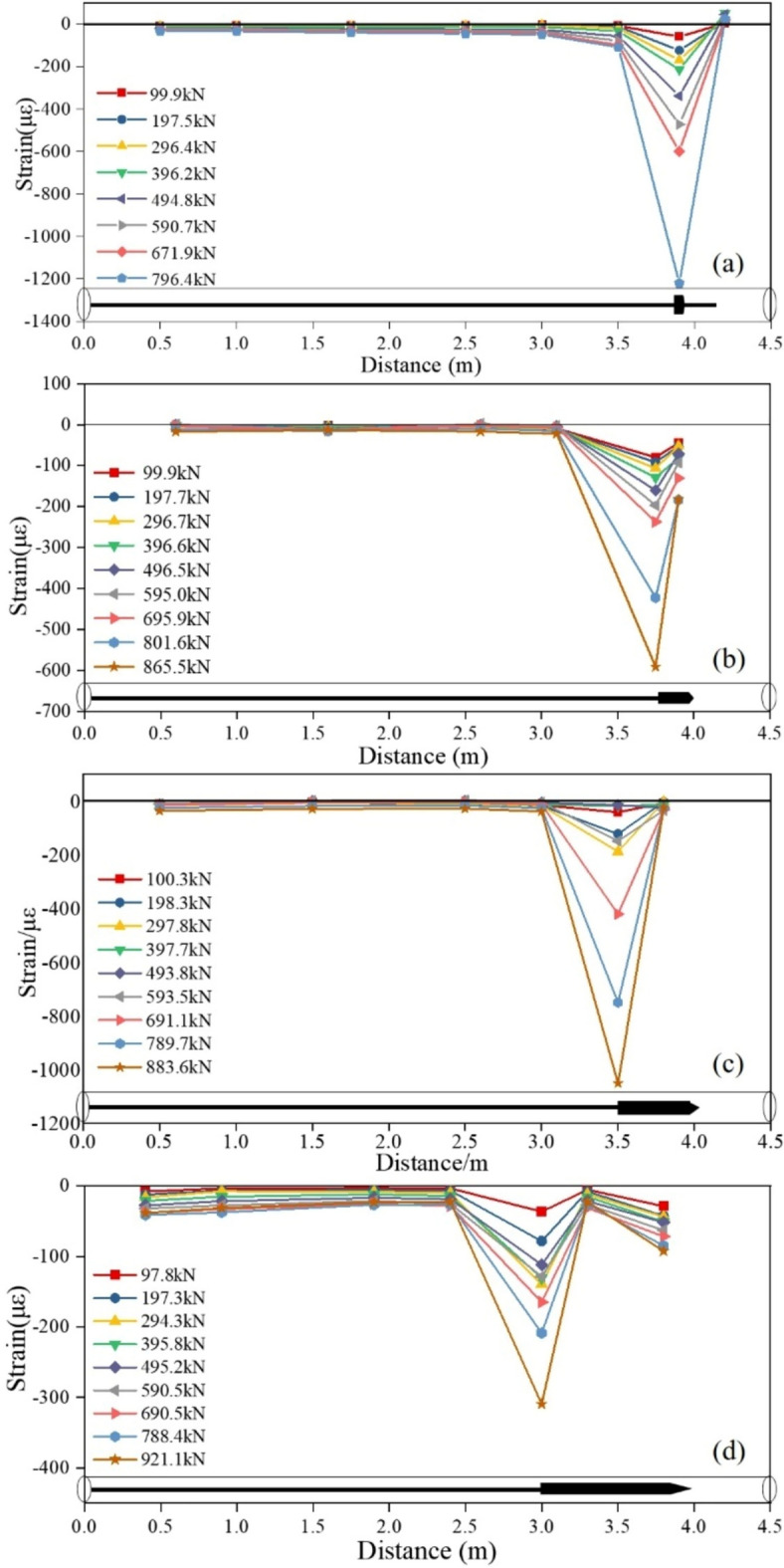
Strain characteristics of anchor cables under different working conditions. (**a**) TPAC; (**b**) PIAH250; (**c**) PIAH 500; (**d**) PIAH 1000.

As shown in Fig. 8, the pressure strain zone was formed in front of the pressure plate of TPAC, while the stress of PIAC was also highly concentrated within a range of about 0.6 m near the bearing plate, reaching its peak at the bearing plate. The maximum strain of the anchor cable increased with the increase of tension load, but the ultimate strains of the four working conditions are -1219.7 × 10^–6^, -1051.7 × 10^–6^, -591.6 × 10^–6^, and -309.5 × 10^–6^, respectively. The maximum compressive strain of the PIAC with a 1000 mm precast anchor head is only 25.37% of that of TPAC, indicating that the strain state of the grout was noticeably improved after the setting the PIAH, and the maximum compressive strain was greatly reduced.

Moreover, a longer PIAH length proves more advantageous in controlling the deformation of the anchor cable. Within the range of 0.5 ~ 0.75 m from the tail of the PIAH to the grout, the pressure strain almost maintained a slight decreasing trend, signifying the predominant anchoring force between the grout and the hole wall. Simultaneously, the PIAH and the annular grout jointly resisted the tensile load, fully exerting the adhesive force between the grout and the hole wall, effectively improving the anchoring force.

## Numerical simulations

### Numerical model

In order to verify the full-scale indoor tension experiments, a numerical model was constructed using finite element method. The numerical simulations in this study were conducted using FLAC3D 5.0 software platform, with hexahedral elements and wedge elements being primarily adopted for discretization. Considering the relatively small dimensions of the PIAH, wedge elements with appropriate mesh refinement were specifically employed to ensure computational accuracy in this critical region. For the surrounding rock mass, conventional hexahedral elements were utilized throughout the model domain. A comprehensive mesh quality check was performed to verify that the element distortion ratios of all discretized components remained within acceptable limits, thereby guaranteeing the reliability of numerical results. The rationale behind this meshing strategy was to achieve an optimal balance between computational efficiency and solution accuracy while properly accounting for the geometric characteristics of different model components.

**Fig. 9:**
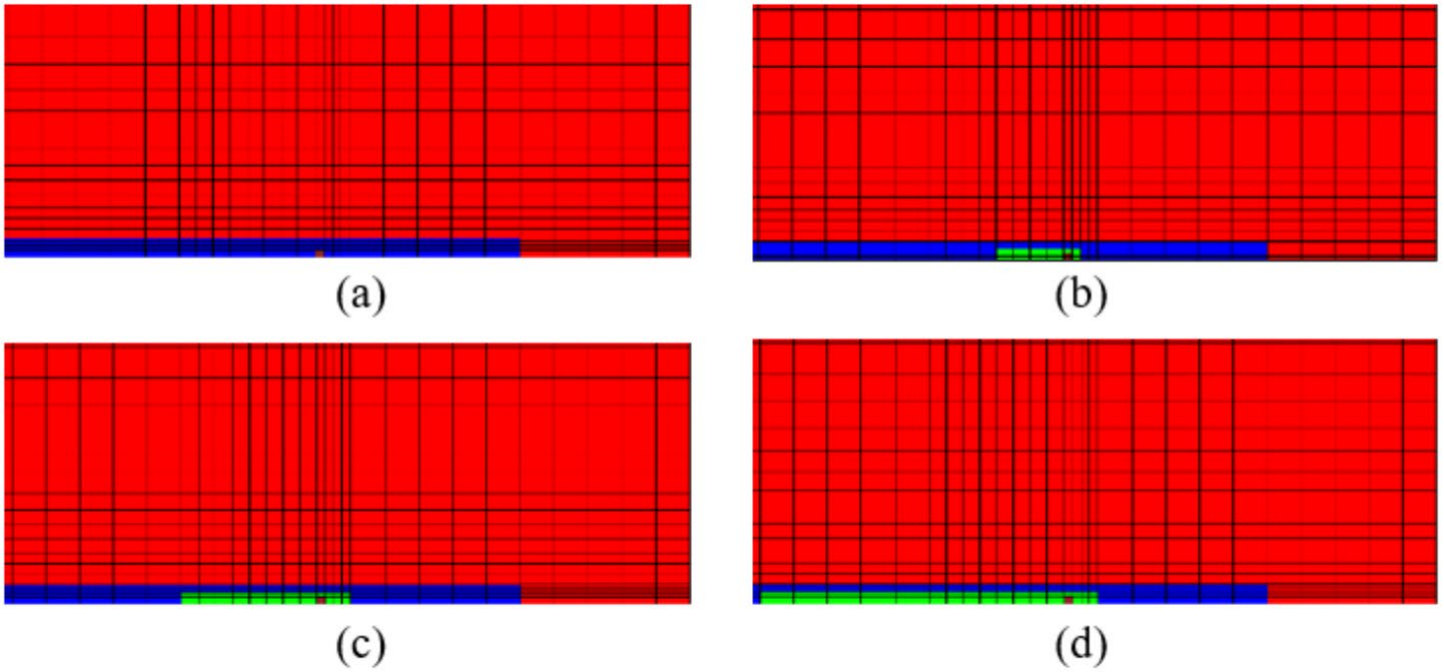
Numerical model of the cable tension simulation with different lengths of precast anchor head. (**a**) G3-1; (**b**) G3-2; (**c**) G3-3; (**d**) G3-4.

The geometric dimensions of the new anchor cable calculation model for precast anchor heads are 5 × 0.8 × 0.8 m, the length of the grout is 4.5 m, and the diameter of the anchor head is 150 mm, which are same with the indoor experiments. In order to improve computing speed, a 1/4 model simulation was conducted based on model symmetry, as shown in Fig. 9. The mesh of the rock mass, grouting, and contact interfaces near the bearing plate are relatively fine, while the mesh rougher in the surrounding rock. The final simulation model has 15,732 elements and 18,287 nodes.

### Simulation parameters

#### Model parameters

The surrounding rock mass, grout, PIAH, and bearing plate are simulated using solid elements. As shown in Table 5, the materials model of the rock mass, grout, and PIAH were set as the Mohr–Coulomb model, while the material model of pressure plate was set as elastic model. The physical and mechanical model parameters for the above materials were consistent with the laboratory testing models. Table 5 also presents the parameter configurations for the interfacial parameters between the steel strand with PIAH, internal anchor head with grout, and grout with surrounding rock. Given that the PIAH, grout, and surrounding rock all made of concrete materials, identical parameters were adopted for the former two interfaces to maintain consistency in material behavior. However, the connection between the steel strand and PIAH was characterized by relatively weaker bonding properties, which was accordingly reflected in the assigned lower cohesion value for this specific interface.

**Table 5:** Physical and mechanical parameter of entity unit.

Model	Unit type	Constitutive model	*E*/GPa	*υ*	*γ*/kg/m^3^	*c*/MPa	*φ*/°
Rock mass	Physical unit	Molar Coulomb	34.5	0.16	2450	7.00	51.99
Grout	Physical unit	Molar Coulomb	25	11.5	2550	7.14	49.96
Bearing plate	Physical unit	Elastic model	210	0.28	7800	–	–
PIAH	Physical unit	Molar Coulomb	34.5	0.16	2450	7.00	51.99
Interface between rock mass and grout	Contact surface unit	–	–	–	–	1.0	49
Interface between PIAH and grout	Contact surface unit	–	–	–	–	1.0	49
Bond strength between steel strand and grout	Contact surface unit	–	–	–	–	0.16	35

#### Parameters of steel strand

The PIAC consists of 4 high-strength low relaxation prestressed steel strand, which has a nominal diameter of 15.24 mm, a standard tensile strength of 1860 kPa and a nominal cross-sectional area of 139 mm^2^. The ultimate bearing capacity of single strand steel strand is 258.5 kN, and the ultimate bearing capacity of 4-strand steel strand is 1034 kN. The elastic modulus of steel strand is 195GPa. The bonding strength of the steel strand is 3.4 MPa, and the bonding force per unit length of a single steel strand can be calculated as 162.28 kN, with a friction angle of 35°. Steel strand unit nodes and pressure plate unit nodes are fixedly connected. The numerical simulation establishes fixed connections between the nodes of the strand elements and bearing plate elements. Prestressing forces are applied as concentrated loads at the strand ends to replicate actual loading conditions. During the simulation process, a stepwise loading protocol is implemented, with the load incrementally increased by 100 kN per loading stage until reaching the maximum design load of 900 kN.

### Calculation conditions

As previously discussed, mirroring the indoor experiments, four distinct specifications of prestressed anchor cables were arranged, comprising the TPAC and three variations of PIAC. The specifics of the four simulated conditions are illustrated in Table 6.

**Table 6:** Simulation working condition of anchor cable.

Condition numbers	Diameter of PIAH /mm	Length of PIAH /mm	The cable form
G3-1	90	0	TPAC
G3-2	90	250	PIAC
G3-3	90	500	
G3-4	90	1000	

### Simulation results

#### Displacement

**Fig. 10:**
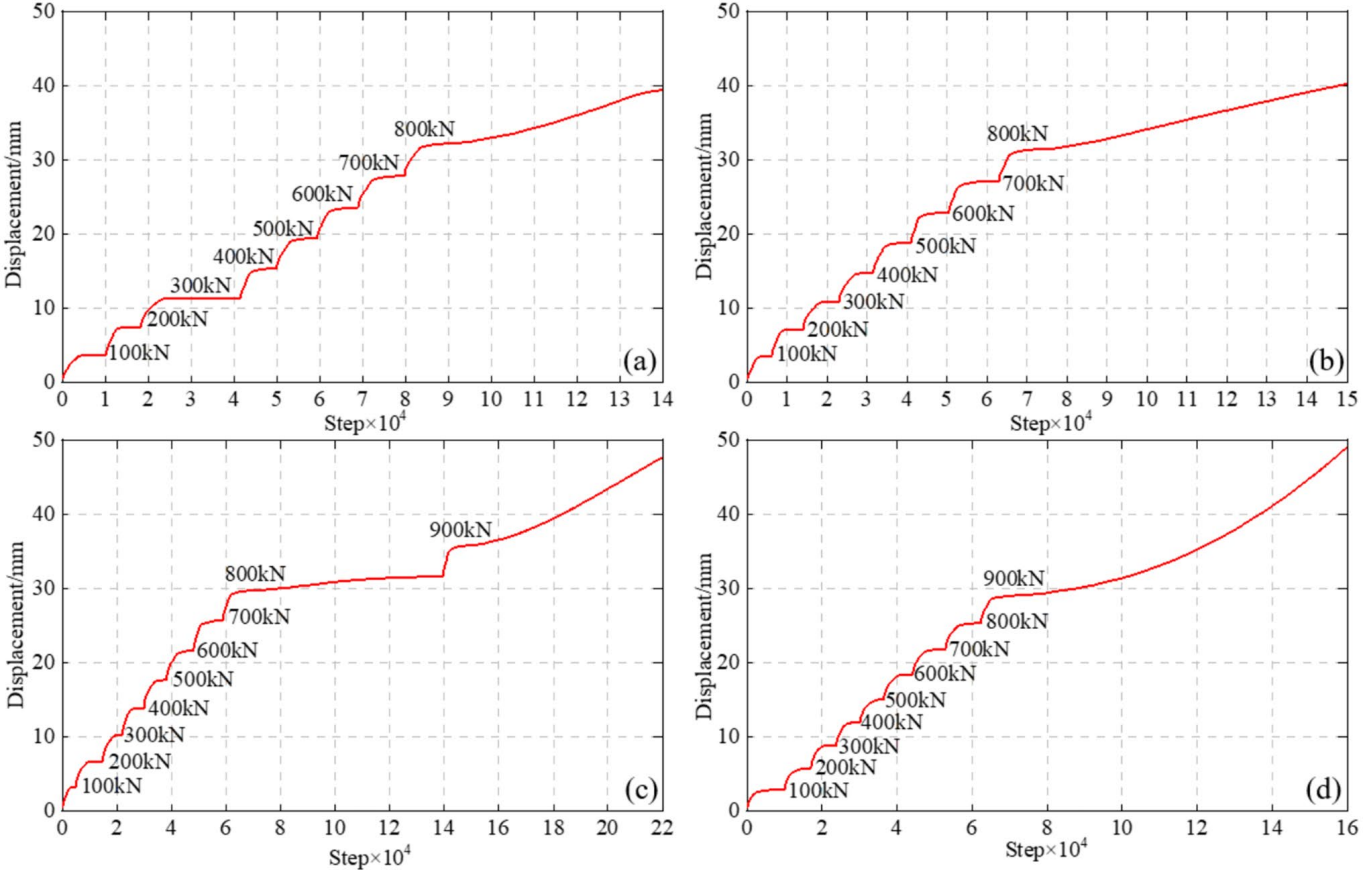
The displacement of anchor cables with varying lengths PIAH under different loads. (**a**) G3-1; (**b**) G3-2; (**c**) G3-3; (**d**) G3-4.

Figure 10 shows the displacement of anchor cables of varying lengths under different loads. The results indicate that under the same conditions, the ultimate anchoring force of the anchor cable increases with the PIAH length. For the G3-1 model with only the support plate and the G3-2 model with a PIAH length of 250 mm, the displacement could steadily increase within the load range of 100 ~ 700kN. When the tension load reached 800kN, the displacement continued to increase and did converge, indicating that the anchor cable was damaged under the 800kN load. For the G3-3 and G3-4 models with the PIAH length of 500 and 1000 mm, the displacement increment remains stable under loads ranging from 100 to 800 kN. When the tension load raised to 900kN, the displacement continues to increase and did not converge, indicating that the anchor cable was damaged under the 900kN load.

**Fig. 11:**
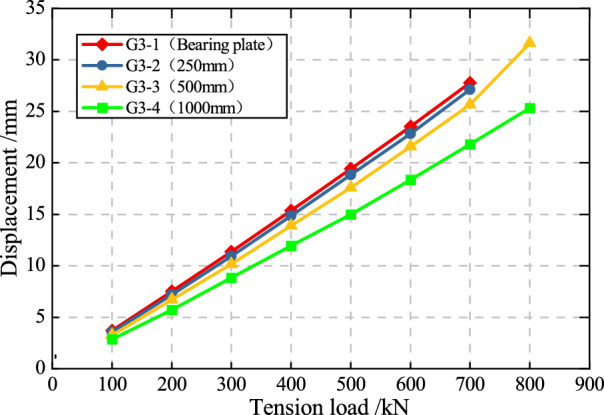
Load**–**Displacement curve of anchor cables in different PIAH lengths.

The relationship between the anchorage displacement of four conditions and the load on each level of anchor cables are shown in Fig. 11. The maximum displacement simulation results of the four groups of anchor cables before failure were 27.9, 27.1, 32.0, and 25.2 mm, respectively, all of which fall within an acceptable range. It can be seen that the displacement of the anchor cable decreases as the PIAH length increases under the same load. Before the anchor cable broke, the displacement of PIAC is smaller than TPAC under same load. Furthermore, the gradient of the curve indicates that the overall elastic modulus of the anchoring system also enhances with the length of the PIAH.

#### The distribution law of axial stress in the grout

**Fig. 12:**
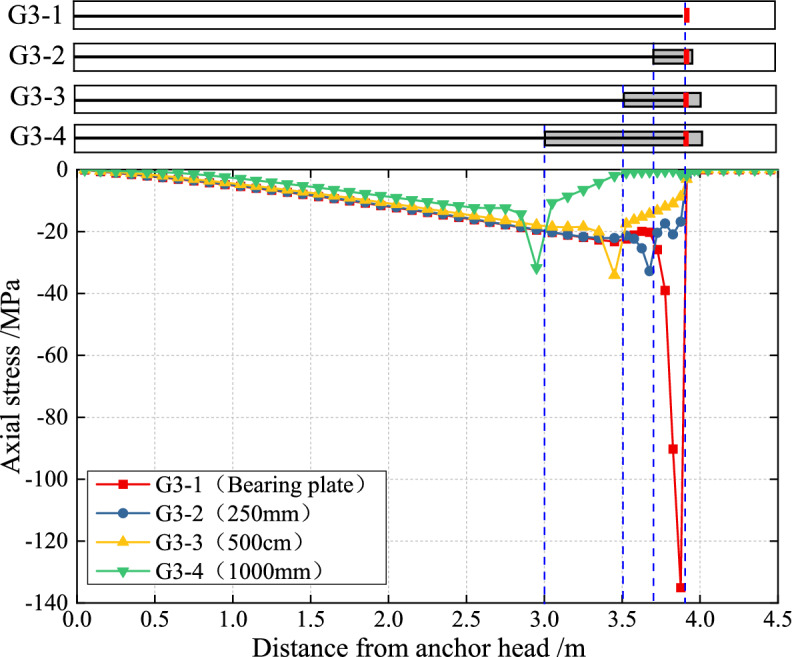
Tension stress distribution of the grout with varying lengths PIAH under the load of 700kN.

Figure 12 shows the effect of the PIAH length on axial stress along the center of the grout under the tension load of 700 kN. The results show that the peak axial stress in the grout was 137.5 MPa and was located at the bearing plate. This concentration of stress is attributed to the TPAC’s precast anchor head having a length of 0 m. The peak axial stress in the grout consistently occurred at the end of the precast anchor head under all three PIAH conditions, irrespective of the internal anchor head length. Moreover, the peak stresses exhibited relatively similar values, ranging from 32.4 to 34.6 MPa, suggesting that the grout’s peak stress is largely independent of PIAH length. In TPAC, the axial stress of the grout at the bearing plate is concentrated, with a peak value approximately 3.5 times higher than that of PIAC. The results imply that compared with TPAC, the use of the PIAH can greatly enhance the anchoring force of the anchor cable.

#### Stress distribution characteristics of the contact surface

**Fig. 13:**
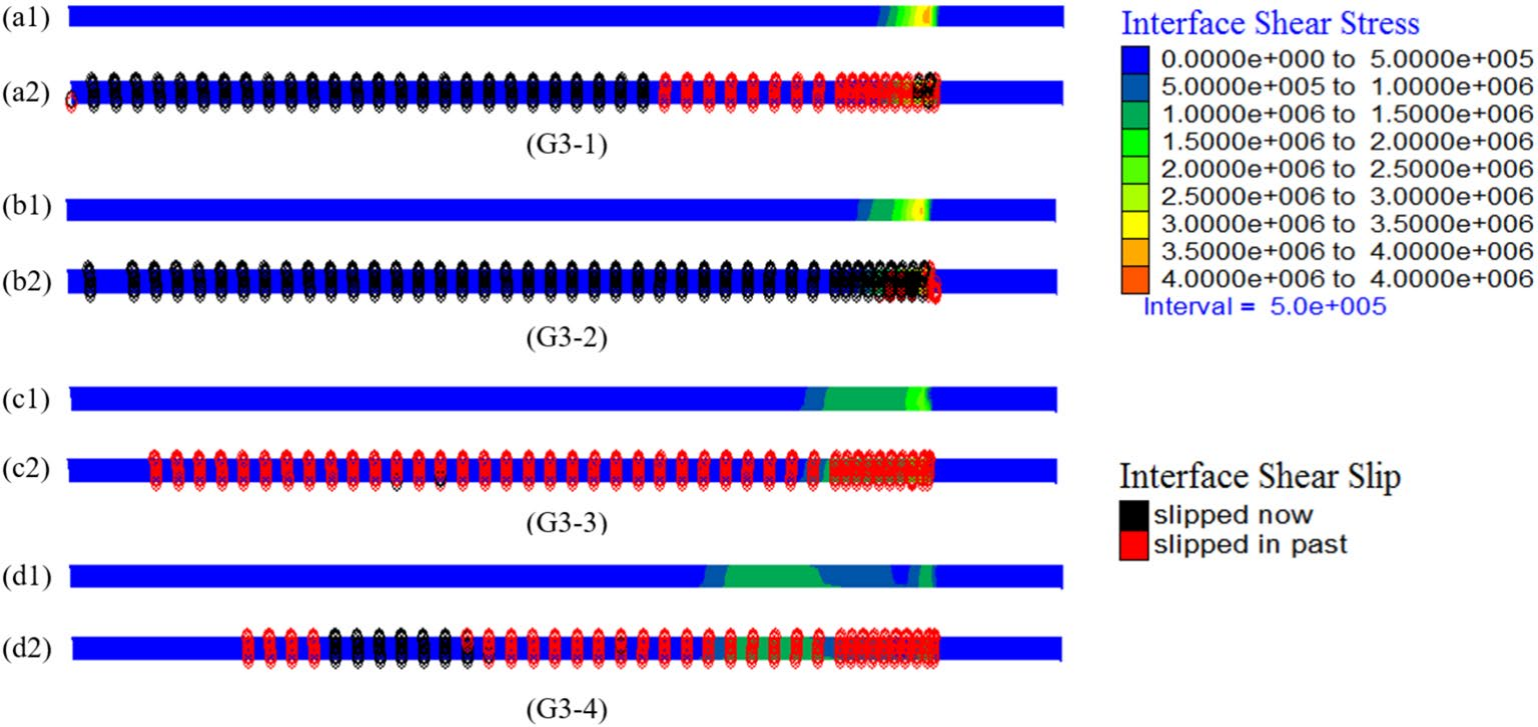
The shear stress distribution of the contact area between the grout and the hole wall of precast anchor heads with varying lengths, and the shear slip state under the load of 700kN.

Figure 13 illustrates the shear stress distribution of the interface between the grout and hole wall of varying lengths PIAH, as well as the shear slip state under the load of 700kN. The results indicate that the interface shear stress can reach approximately 4 MPa and is concentrated near the bearing plate in the absence of a PIAH. In contrast, after installing the PIAH, the shear stress is primarily distributed along the PIAH, with a significant reduction in stress concentration. Therefore, the length of the shear slip zone at the interface between the grout and the hole wall increases as the length of the PIAH decreases, accompanied by a reduction in the degree of shear stress concentration on the contact surface.

**Fig. 14:**
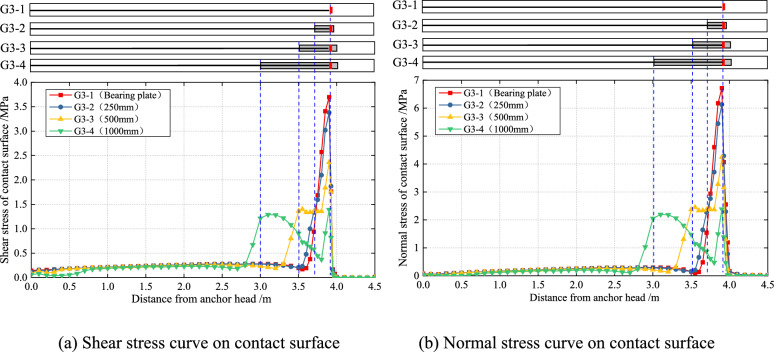
Stress curves of the contact area between the grout and the hole wall of precast anchor heads of varying lengths under a load of 700 kN.

Figure 14 shows the distribution of shear stress and normal stress between the grout and hole wall of varying lengths PIAH under the tension load of 700 kN. The results show that the peak values of shear stress and normal stress at the interface between the grout and the hole wall increase as the length of the PIAH decreases. The maximum shear stress increased from 1.35 to 3.7 MPa, while the maximum normal stress rose from 2.3 to 6.8 MPa, and the stress concentration phenomenon becomes more obvious while the distribution range becomes smaller. The shear stress and normal stress on the interface in TPAC are more inclined to concentrate because it does not have PIAH. The results indicate that the stress state of the three types of PIAC has been greatly improved compared with TPAC. The length of the PIAH has no effect on the positions of peak indirect contact shear stress and normal stress between the grout and the hole wall. The positions of peak shear stress and normal stress for the four types of anchor cables are basically located on the contact surface between the grout and the hole wall at the load-bearing plate.

#### The yielding state of the grout at the end of PIAH

**Fig. 15:**
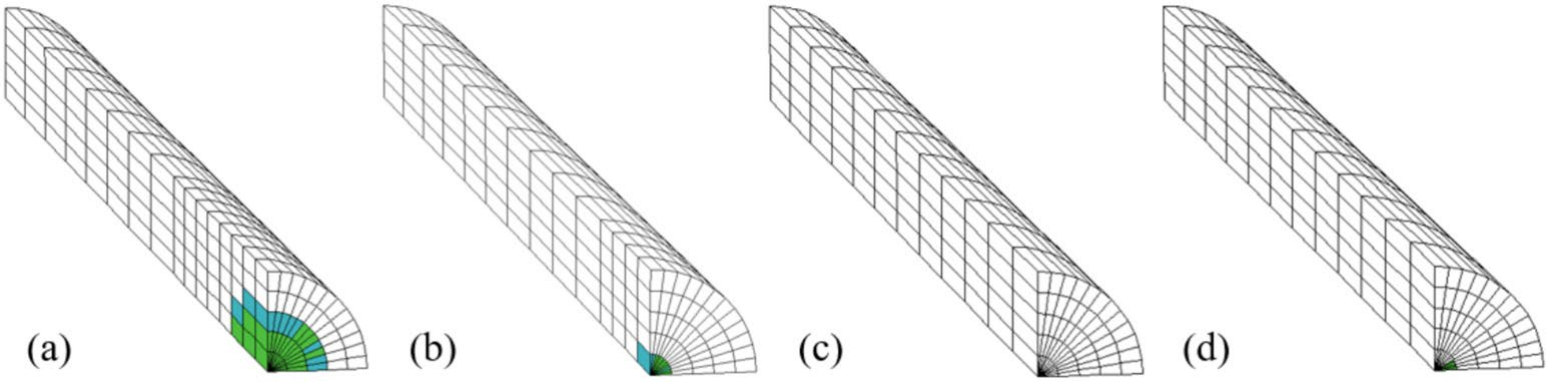
The shear yield condition of grout at the end of the PIAH under a load of 700kN. (**a**) G3-1; (**b**) G3-2; (**c**) G3-3; (**d**) G3-4.

The yield state of varying length load-bearing plates or precast anchor head front grouting under a tension load of 700kN is shown in Fig. 15. The length of the PIAH has a significant impact on the yield state of the grout. The shorter the length of the PIAH, the larger the range of yielding strength of the grouting. Due to the absence of the PIAH in TPAC, the yielding range and degree of the grout in front of the bearing plate are the largest and most severe.

**Fig. 16:**
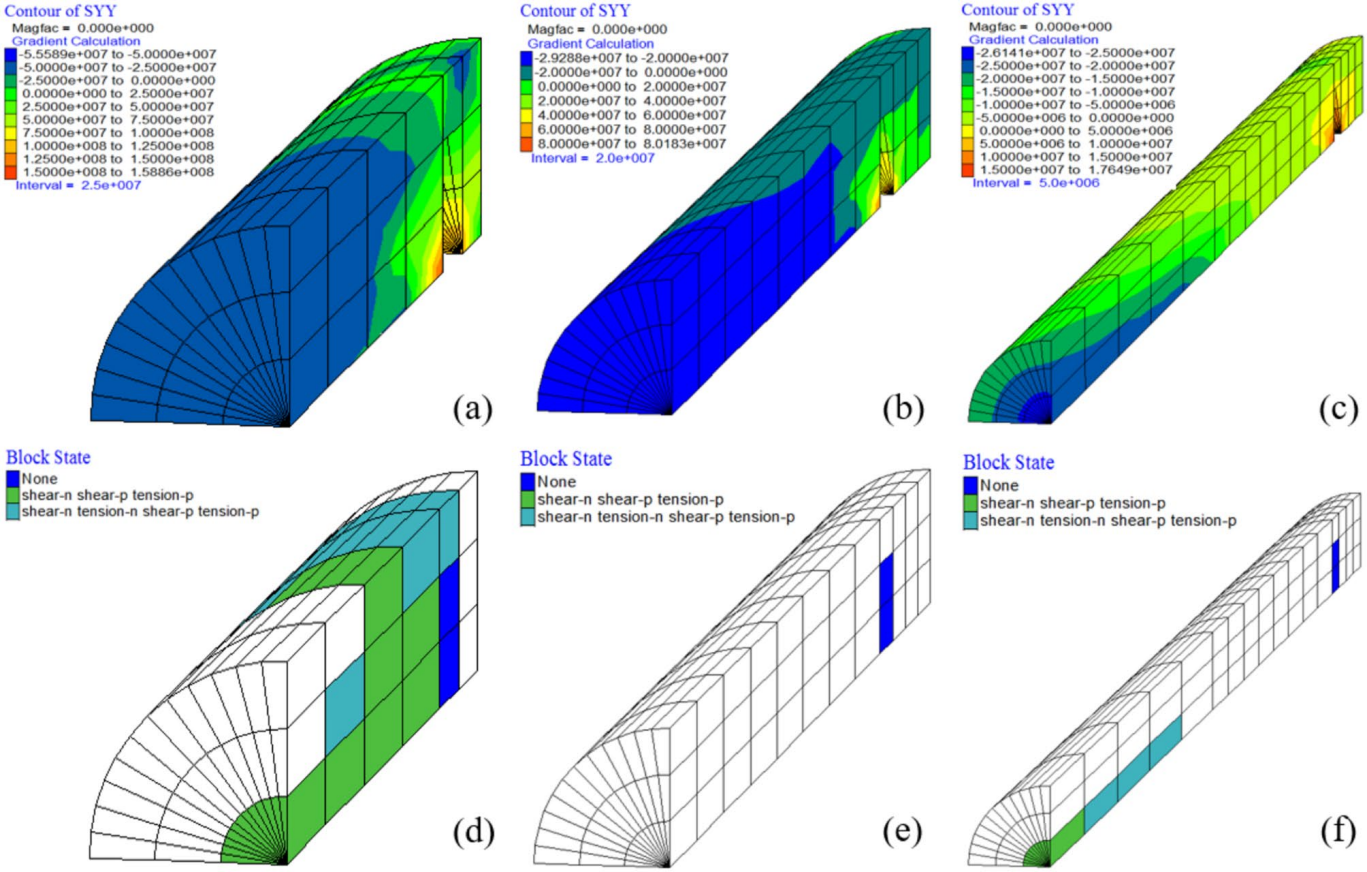
The yield state of PIAH under a tensile load of 700 kN. (**a**) G3-2; (**b**) G3-3; (**c**) G3-4.

Figure 16 shows the yield state of PIAH under a tensile load of 700kN, and the blue area in the figure represents the pressure plate. As shown in Fig. 16a–c, the maximum axial stresses (SYY) of the three groups of the PIAH are measured at 158, 82, and 17.6 MPa, respectively. The result indicates that longer PIAH lengths lead to a reduced area of shear yielding within the PIAH, with the severe yielding state reflecting concentrated stress distribution. At the same time, the shear yield area of PIAH decreases with longer PIAH, and the stress concentration increases with the yield degree weaken (Fig. 16d–f).

## Discussion

### Relative errors between experimental and simulation results

Although the design of experimental groups and material parameters in above numerical simulations was largely consistent with the full-scale physical model tests, certain relative errors were observed in the results. For instance, the ultimate bearing capacities obtained from the four physical model tests were 796.4, 865.5, 883.6, and 921.2 kN, respectively. In contrast, the numerical simulations produced ranges of values 700 ~ 800, 800 ~ 900, 800 ~ 900, and 800 ~ 900 kN as shown in Fig. 10 due to the difficulty of applying load in 1 kN increments given the considerable time required for convergence in each calculation. It can be observed that the physical test results all fall within the ranges obtained from numerical simulation, demonstrating a good agreement, with only a minor deviation in the 1000 mm-PIAH group, possibly due to the loading gradient set in the simulation (the increment of applied load is 100 kN per time). Additionally, the displacements of the anchor cables at ultimate load in the four physical tests were 65.2, 47.6, 49.1, and 55.4 mm, respectively, while the numerical simulations yielded corresponding values of 27.9, 27.1, 32.0, and 25.2 mm. Although the failure displacement of the anchor cables in both datasets shows weak correlation with the length of the PIAH, the physical test results vary within a range of 47.6 ~ 65.2 mm, whereas the numerical results vary within a narrower range of 25.2 ~ 32.0 mm. This discrepancy may be attributed to insufficient grout filling during the preparation of the physical specimens, which falls within an acceptable margin of error for numerical simulations. In future studies, increasing the number of test groups may help reduce such relative errors.

### Maximum effective length of PIAH

**Fig. 17:**
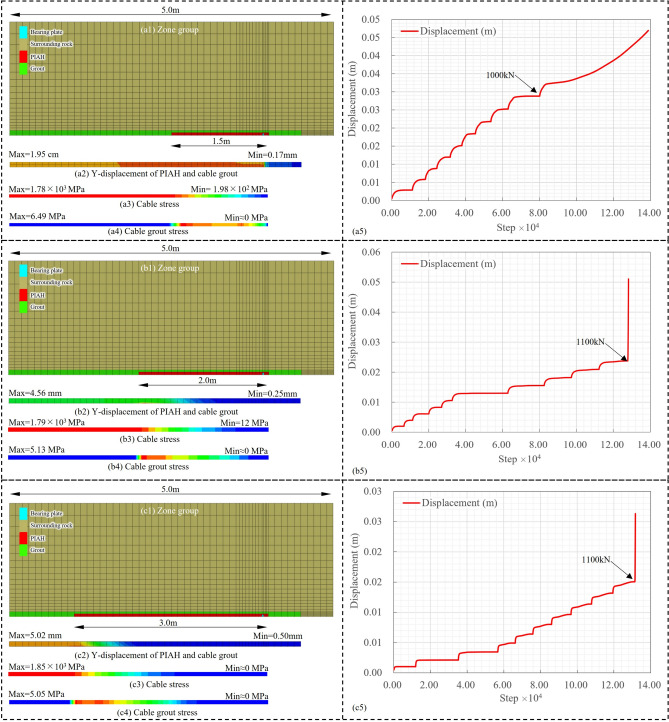
Numerical simulation results of anchor cable stress and ultimate bearing capacity characteristics under different PIAH lengths. (**a1** ~ **a5**) PIAH length: 1.5m; (**b1** ~ **b5**) PIAH length: 2m; (**c1** ~ **c5**) PIAH length: 3m.

The aforementioned experimental and simulation results support our conclusion that the ultimate bearing capacity is proportional to the length of the precast inner anchor head (PIAH), yet they do not indicate the existence of a maximum effective length or whether further increasing the length would lead to diminishing returns. To address this question, we redesigned three sets of experiments with PIAH lengths of 1.5, 2, and 3 m, using the following control indicators: anchor cable displacement, cable stress, grout stress, and displacement-step curves (Fig. 17a1,b1,c1).

The results show that the maximum displacement of the grout still occurs at the interface between the PIAH and the strand (Fig. 17a2,b2,c2). Although increasing the PIAH length reduces the displacement of the grout (from a maximum of 1.95 cm down to 4 ~ 5 mm), the cable stress distribution (Fig. 17a3,b3,c3) indicates that the stress in the anchor cable is primarily concentrated within approximately 1 m beyond the connection point, gradually decreasing to zero toward the end of the anchoring segment. This behavior is consistent with observations reported by many researchers, indicating that the stress along the anchor cable follows a pattern of initial increase followed by a gradual decrease^30^. Correspondingly, the stress in the grout also exhibits a similar decay trend toward the end of the anchored section (Fig. 17a4,b4,c4).

Regarding the core issue of ultimate bearing capacity, it is observed that when the PIAH length reaches 1.5 m, the ultimate bearing capacity can reach about 1000 kN (Fig. 17a5). However, when the PIAH length reaches 2 m and further increases to 3 m, although the displacement of the anchor cable decreases under each loading step, the maximum bearing capacity shows almost no obvious improvement (Fig. 17b5,c5). Moreover, under the 2 and 3 m PIAH conditions, a sudden sharp increase in cable displacement occurs when a load of 1100 kN is applied, suggesting possible abrupt structural failure.

Based on these numerical simulation results, we conclude that for the given test conditions (total anchor cable length of 4 m), the maximum effective length of the PIAH likely lies between 1.5 and 2 m. Beyond a PIAH length of 2 m, no substantial enhancement in the ultimate pull-out resistance can be expected, and the stress concentration may shift toward the anchor head region. It should be noted, however, that these conclusions are derived solely from numerical simulations, and more accurate validation awaits further physical model experiments.

### Geotechnical applicability of PIAC

In practical engineering, anchor cables are often applied not only in hard rock but also in soft rock with poor rock mass quality. Based on this, we investigated the applicability of the PIAC in different rock strata using numerical simulation methods. First, two numerical simulation schemes were designed for hard rock (sandstone) and soft rock (mudstone), respectively. The parameters of the grout and the PIAH remained unchanged with G3-4 in both models, while the surrounding rock parameters were set based on assumed values, and the properties of the interface between the surrounding rock and the grout were adjusted accordingly.

**Fig. 18:**
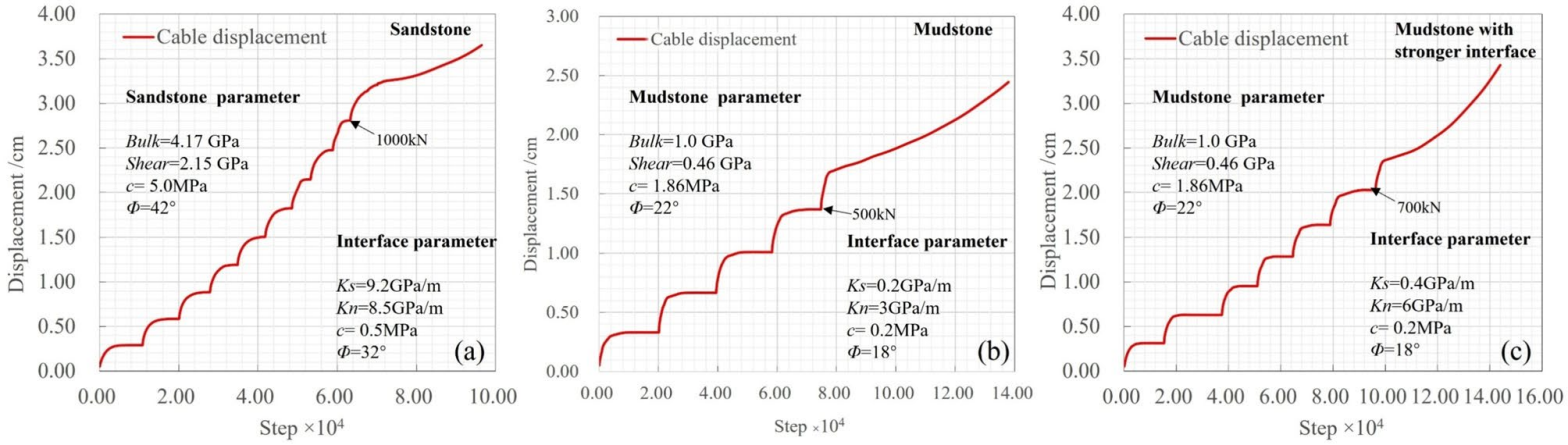
Numerical simulation results of load–displacement curves of PIAC in different rock and soil masses. (**a**) Sandstone; (**b**) Mudstone; (**c**) Mudstone with stronger interface.

The results indicate that when the surrounding rock is sandstone (hard rock), the ultimate bearing capacity shows little change and can still reach nearly 1000 kN (Fig. 18a). However, when the surrounding rock is mudstone, the ultimate bearing capacity is only about 500 kN (Fig. 18b), which suggests that in weaker surrounding rock, the anchoring capacity cannot be fully utilized.

Given the relatively low strength of mudstone, an additional simulation was conducted (keeping the mudstone properties and other conditions unchanged while only increasing the strength of the structural interface) to examine the influence of the interface strength between the surrounding rock and the grout on the ultimate bearing capacity. As shown in Fig. 18c, after doubling the strength parameters (*k*_*n*_ and *k*_*s*_) of the interface, the ultimate bearing capacity of the anchor cable increased to approximately 700 kN.

These results demonstrate that replacing hard rock with concrete has a limited effect on the ultimate bearing capacity, which also validates the reliability of the previous experimental design. Furthermore, when PIAH is used in soft rock conditions, enhancing the strength of the interface between the soft rock and the grout can improve the geotechnical applicability of the PIAC structure.

### Limitations and future research directions

The experimental investigation in this paper was conducted in a semi-enclosed laboratory setting to validate the structural performance of the PIAC structure and determine the optimal length of the precast internal anchor head. It should be noted that the surrounding geomaterial was simulated using cast concrete and lacking of long-term monitoring, which inherently limited the consideration of factors such as variations in soil moisture conditions, dynamic loading scenarios, and long-term material aging effects. Consequently, the experimental conditions deviated to some extent from actual engineering environments. Building upon the findings of this study and complementary numerical simulations, future work will focus on designing more diversified testing protocols to enhance the geotechnical applicability of PIAC structures. By integrating advanced monitoring techniques, we aim to quantitatively elucidate the influence of key factors including anchor head length, material properties, and structural configuration on the anchoring performance of PIAC structures, thereby providing more reliable guidance for engineering practice.

## Conclusions

The paper introduced a novel Anchor cable with precast internal-anchor-head (PIAC) structure and outlined its design calculation methodology via theoretical calculations. The efficacy of the structure was validated through comprehensive full-scale tension experiments and numerical simulations. The conclusions drawn from the study are as follows:

1. By pouring the grout and the columnar grout of the upper anchoring section together, the anchoring force of TPAC can be sequentially transmitted from the unbound steel strands to the steel bearing plate, high-strength precast concrete anchor head, and the joint between the grout and the surrounding rock, greatly improving the stress state of the anchoring system.
2. Compared with TPAC, PIAC can fully exert the bonding force between the grout and the hole wall, noticeably improve the strain state of the grout, and increase its ultimate bearing capacity from 796.4 to 921.2kN. The ultimate strain can be reduced from −1219.7 × 10^–6^ to −309.5 × 10^–6^, a decrease of 74.63%. Moreover, longer precast internal-anchor-head (PIAH) are more favorable for controlling cable deformation.
3. Under identical conditions, the ultimate anchoring force of the anchor cable increases with an increase in length of PIAH, thereby enhancing the overall elastic modulus of the anchoring system. Compared to the grout of the TPAC, the degree of axial stress concentration of PIAC was merely 28.57%, effectively mitigating the stress concentration. Furthermore, the anti-shear slip capability of PIAC was substantially improved, resulting in a reduced yield area of the grout.
4. The maximum effective length of PIAH is about 1.5 ~ 2m under the test conditions of a 4m PIAC and further length increases produce diminishing returns. Meanwhile, PIAC can be used not only in hard rock formations, but also in soft rock formations after improving the strength of the interface between the surrounding rock and the grout.

## Data Availability

The data used in this study are available from the corresponding author upon reasonable request.
